# Endoscopic repair of a perforated duodenal ulcer: time to close the gap

**DOI:** 10.1016/j.vgie.2024.01.006

**Published:** 2024-02-05

**Authors:** Andrew Canakis, Shayan S. Irani

**Affiliations:** 1 Division of Gastroenterology and Hepatology, University of Maryland School of Medicine, Baltimore, Maryland; 2 Division of Gastroenterology and Hepatology, Virginia Mason Medical Center, Seattle, Washington

## Abstract

Video 1 Management of an acute perforated duodenal ulcer.

Management of an acute perforated duodenal ulcer.

## Introduction

Although penetrating duodenal ulcer perforations have become rare in the era of proton pump inhibitors, they can be difficult to manage, with an associated mortality ranging from 8% to 25%.^1^ Endoscopic options include through-the-scope clips, over-the-scope clips, and self-expandable metal stents. Choosing a closure method is often dependent on the perforation’s location, and size and the operator’s experience.^2^ Recently, the application of through-the-scope suturing has emerged as a versatile option for closing full-thickness perforations and fistulas, but not much is published on perforated duodenal ulcers.^3^, ^4^, ^5^, ^6^ Endoscopic suturing is superior to standard clips, as it allows for adequate robust suturing with an airtight closure in a short amount of time.^7^^,^^8^ The challenges associated with a perforated ulcer are contamination of the retroperitoneum and/or peritoneum and the fibrotic nature of ulcers that can preclude approximation of the margins. However, when the surgical risks are prohibitive, management with drains and endoscopy may be required. In this case, we present the management of an acute perforated duodenal ulcer that was repaired endoscopically (Video 1, available online at www.videogie.org).

## Case

A 70-year-old man with a history of arthritis (on daily ibuprofen), alcohol use disorder, and multiple abdominal surgeries, including diverticulitis status post–Hartmann’s pouch complicated by a colovesical fistula with subsequent colostomy reversal and an abdominal wall mesh, was transferred with concern for an acute duodenal perforation. Five days prior he developed progressive abdominal pain, fever, nausea, vomiting, and inability to tolerate oral intake. A CT scan of the abdomen revealed free air and fluid near the duodenum concerning for duodenal perforation secondary to nonsteroidal anti-inflammatory drug use. He was started on broad-spectrum antibiotics. A percutaneous drain was placed locally, and he was transferred to our center for further care, where he was also deemed a poor surgical candidate.

**Figure 1:**
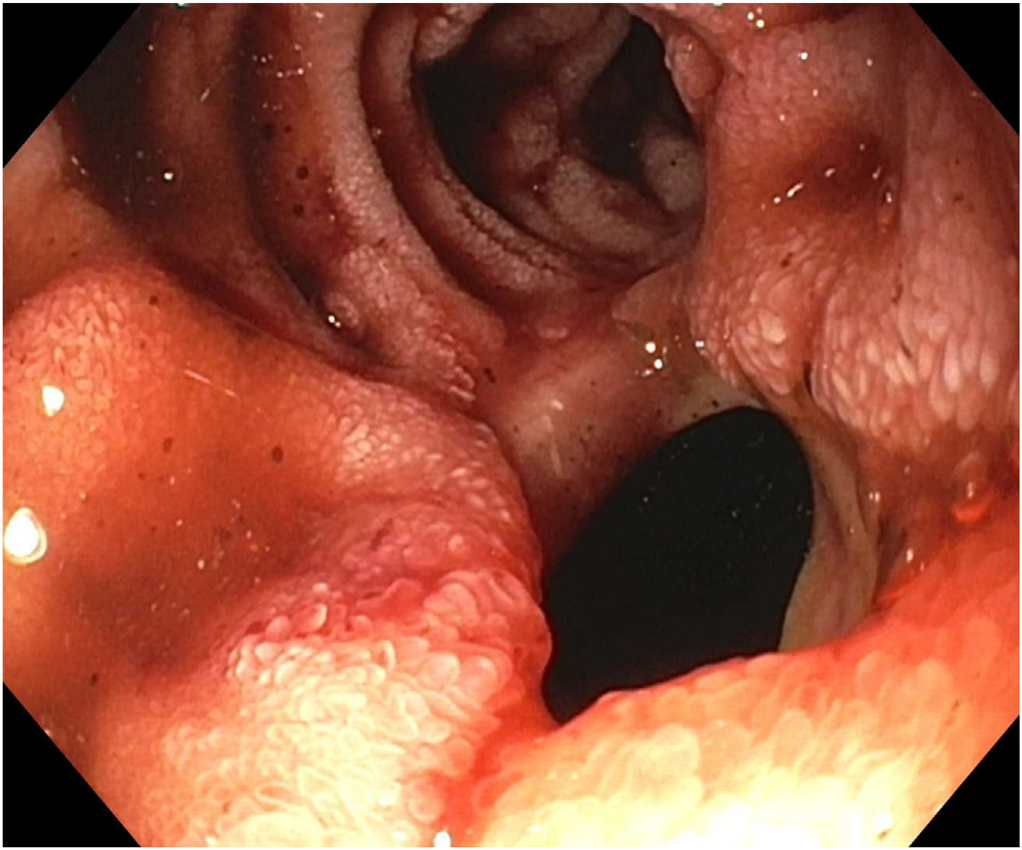
Endoscopic view of 1-cm duodenal perforation.

**Figure 2:**
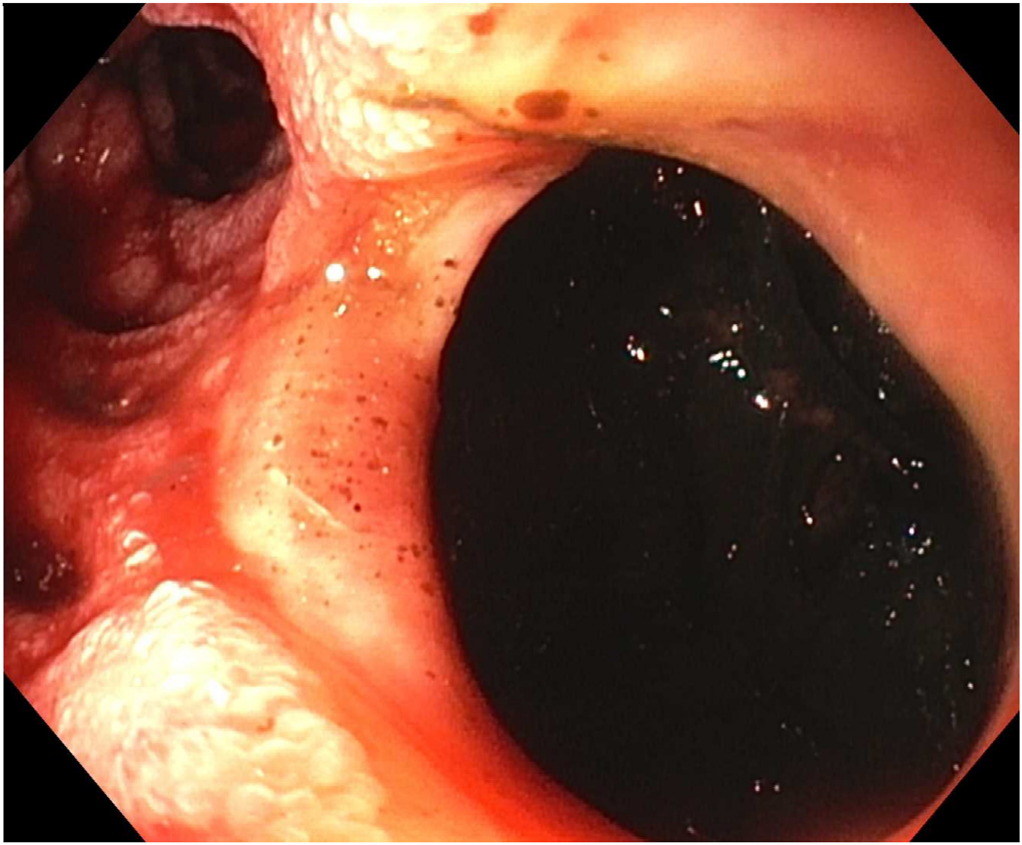
Endoscopic view of 1-cm duodenal perforation.

**Figure 3:**
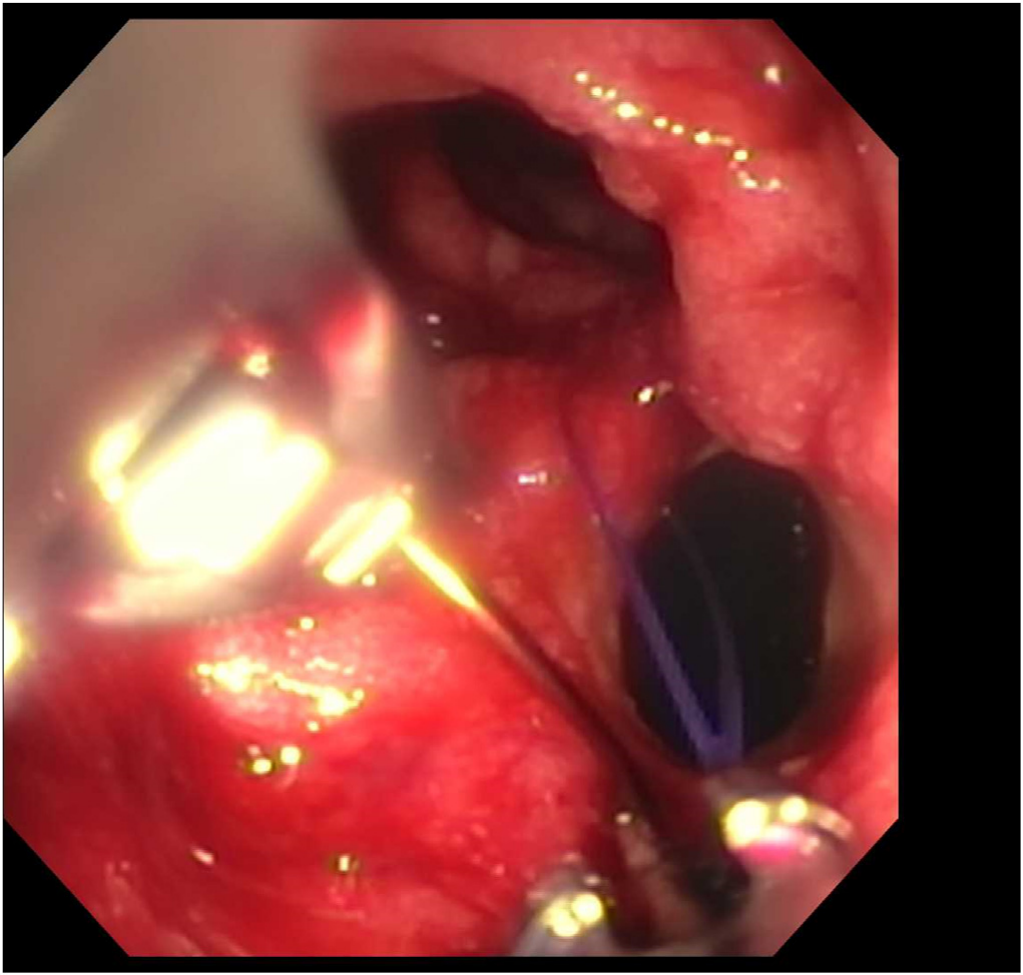
Full-thickness suturing of the perforation.

**Figure 4:**
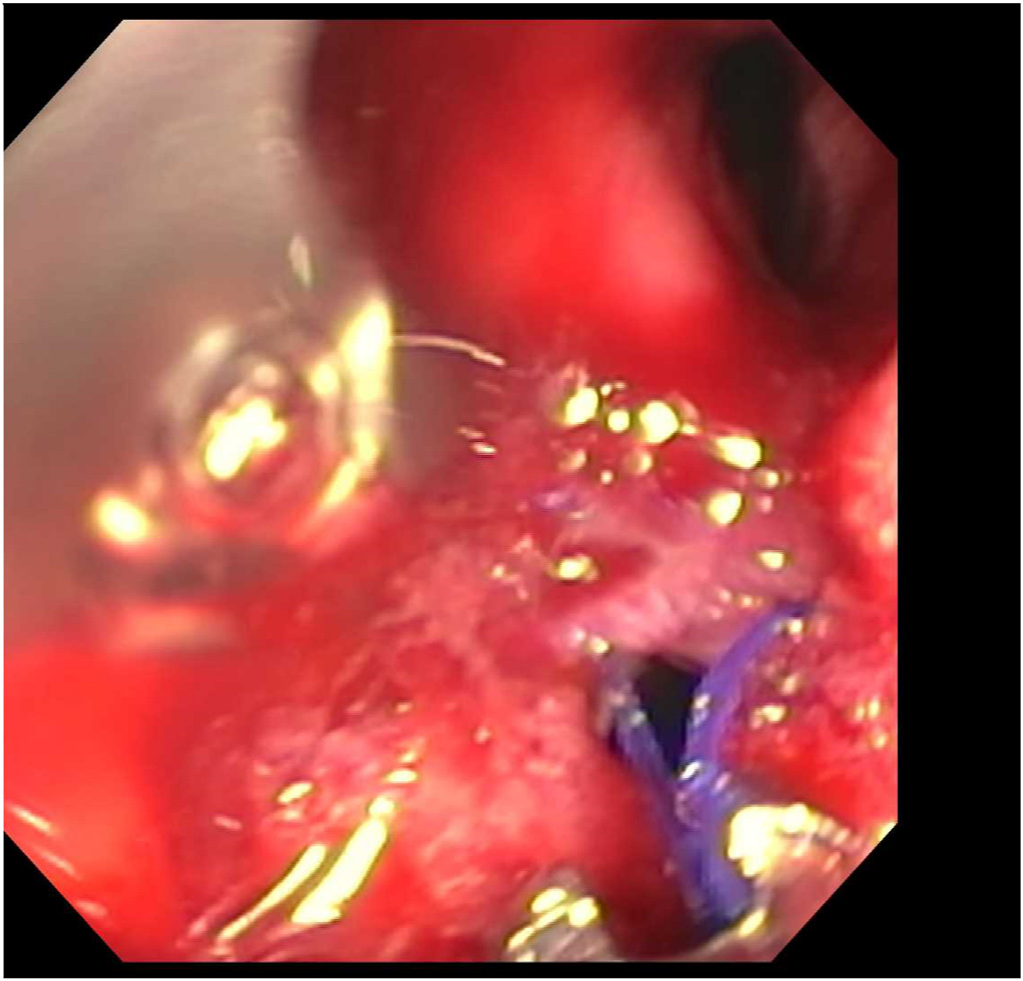
Full-thickness suturing the perforation.

**Figure 5:**
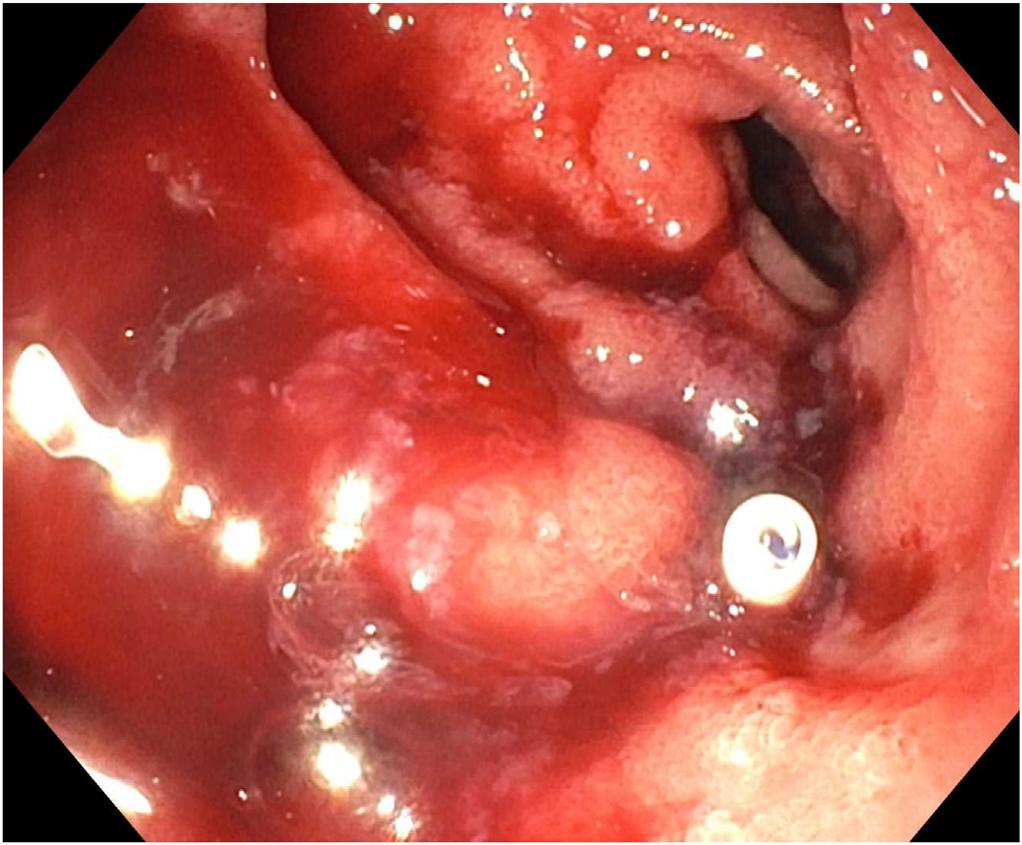
Cinch deployed with closure of defect.

Endoscopy demonstrated a 1-cm duodenal perforation with severe duodenitis with several shallow ulcers through the duodenal loop (Figs. 1 and 2). The location and size of the perforation seemed amenable to endotherapy. An over-the-scope clip was not used due to concerns that if a recurrent leak occurred around the over-the-scope clip, it would be harder to fix and require removal, which can sometimes be challenging. A through-the-scope suture device was not used, as we aimed for full-thickness closure. A full-thickness suturing device (OverStitch Endoscopic Suturing System; Apollo, Austin, Tex, USA) was used to place a single running suture to close the perforation (Figure 3, Figure 4, Figure 5). This was accomplished with an out-to-in bite distally into the defect, then bringing that to the contralateral wall with an in-to-out bite (with at least 3-4 mm of healthy tissue on either side grasped). Of note, the procedure was done under general anesthesia, which allowed manipulation of the double-channel therapeutic scope more comfortably in a long scope position, as is needed sometimes in the duodenum. Finally, closure was confirmed with contrast injection. A nasojejunal tube was placed and used for enteral nutrition. Duodenal biopsies were benign, and he had a normal gastrin level. Note, percutaneous drainage was important and effective in not only draining the leaked contents but also perhaps in reducing duodenal edema from the bilio-pancreatic juices, allowing for easier suture closure.

**Figure 6:**
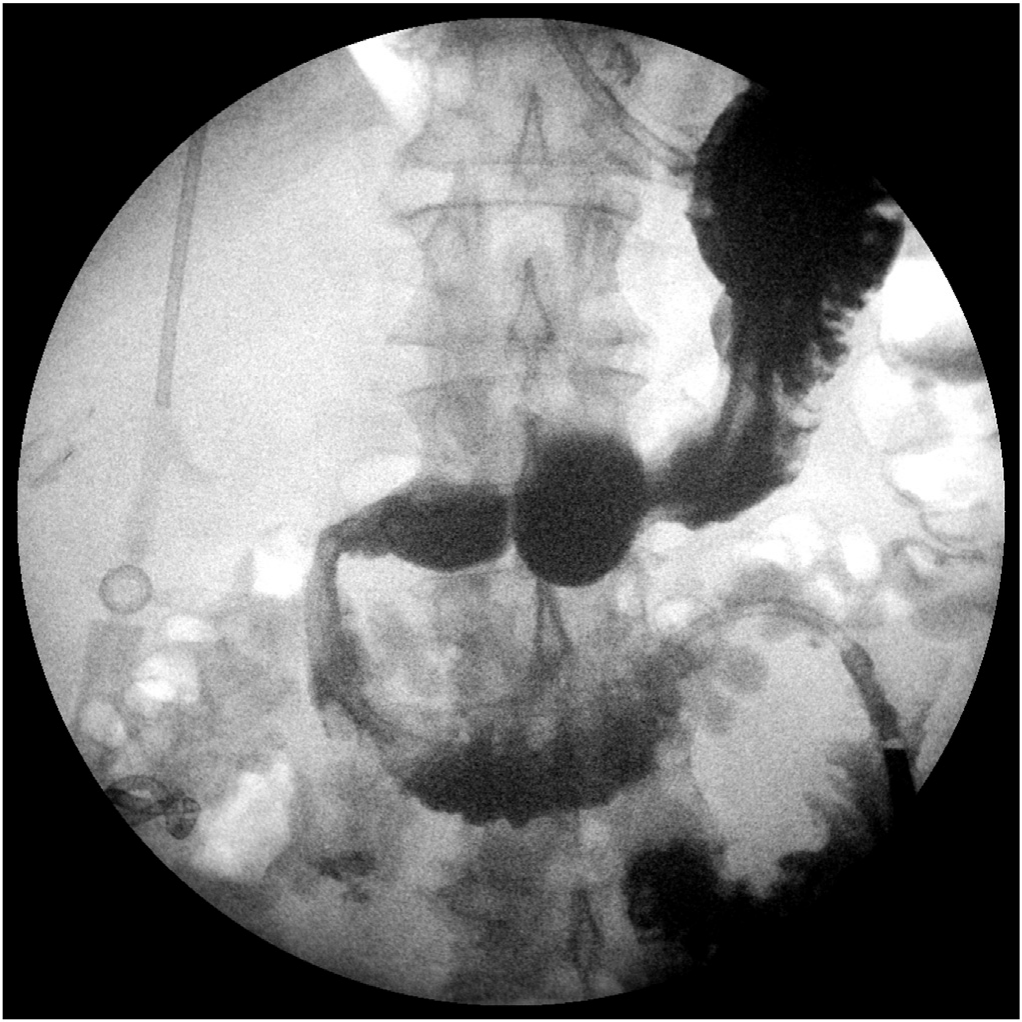
Upper GI series on postprocedure day 6.

**Figure 7:**
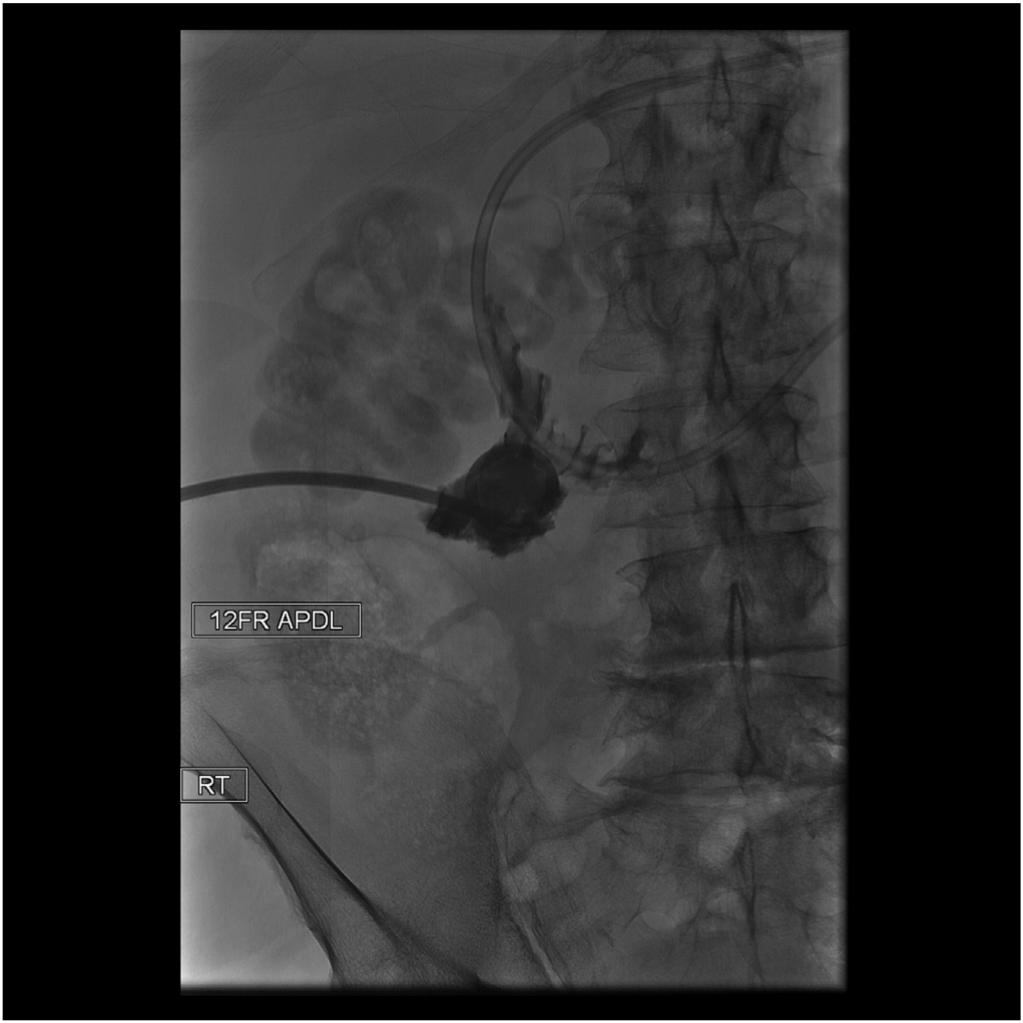
Tube check with contrast injection demonstrated a small persistent connection to the duodenum.

**Figure 8:**
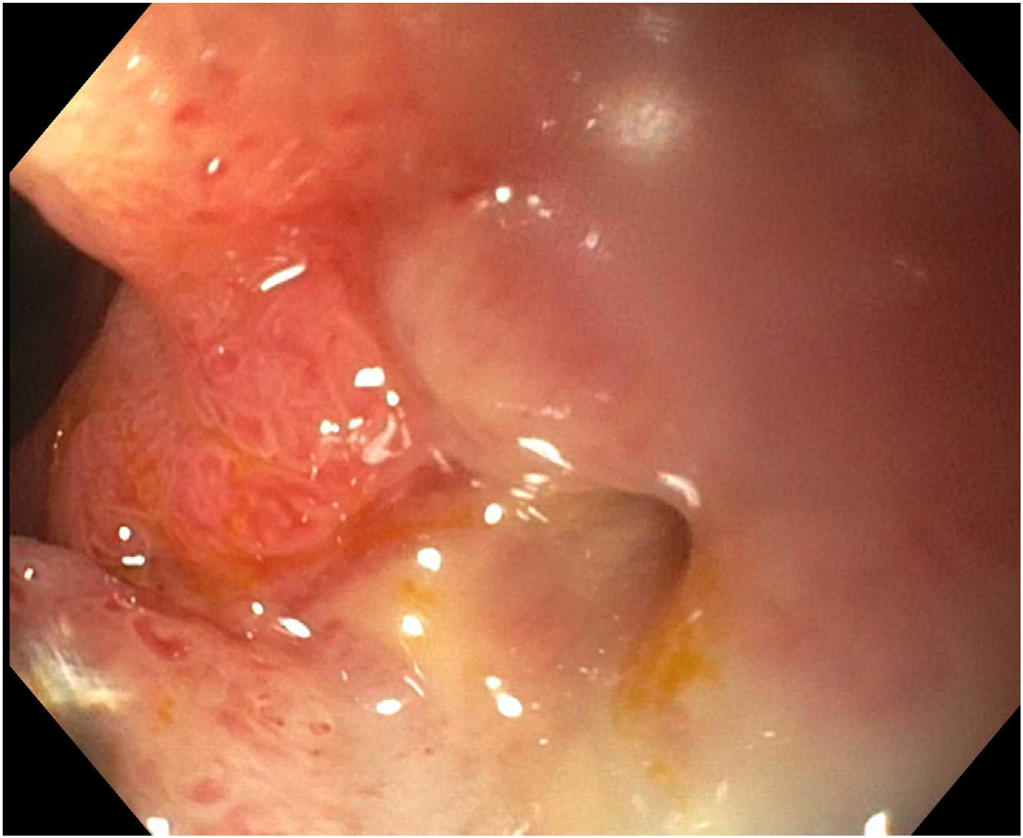
Second endoscopy showing a small residual perforation.

**Figure 9:**
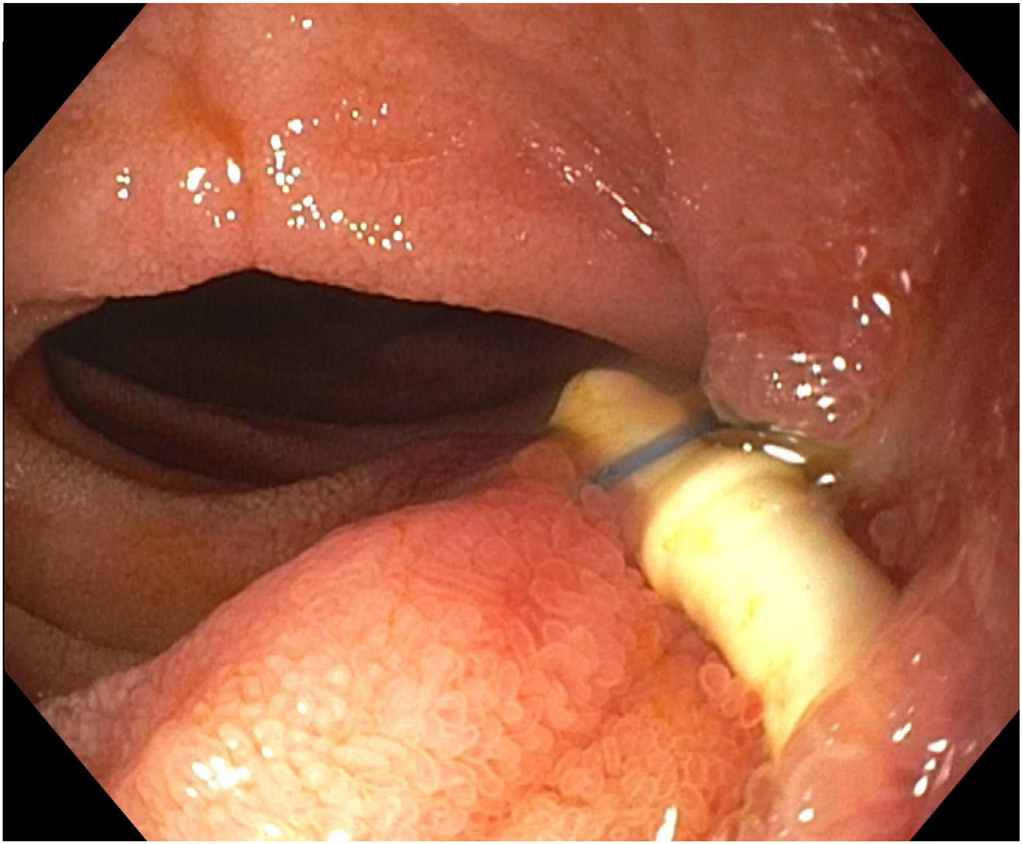
Second endoscopy with another full-thickness suture placed.

The patient’s abdominal pain improved. An upper GI (UGI) series on postprocedure day 1 showed no leak, and he was started on clear liquids. Repeat UGI series on day 6 and CT scan on day 7, as is our practice with contaminated perforations, also showed no leak of oral contrast (Fig. 6). However, a tube check with contrast injection demonstrated a small persistent connection to the duodenum (Fig. 7). Consequently, a second EGD with another full-thickness suture was successfully performed (Figs. 8 and 9). His drain was observed for an additional 4 days with no evidence of a leak, and the drain was removed on day 11 after confirming no fistula on tube check and resolution of the abscess. He was then advanced to a full liquid diet the following day with guidance to advance to a regular diet over the next 3 days and discharged on day 12. Antibiotics were administered for a total of 14 days. He has had no recurrence of abdominal pain or UGI symptoms for the last 7 months and has remained off nonsteroidal anti-inflammatory drugs.

## Conclusion

In a high-risk surgical patient, endoscopic full-thickness suturing could be used to repair even a perforated ulcer with peritoneal contamination along with an external drain placement. Checking on the integrity of the sutures not only the next day, but also 5 to 7 days after closure, is important, given the risk of dehiscence due to the contaminated and possibly unhealthy tissue. This can be readdressed with suturing in the right setting.

## Disclosure

Dr Irani is a consultant for Boston Scientific and Gore. Dr Canakis disclosed no financial relationships relevant to this publication.

## Supplementary data

Video 1 Management of an acute perforated duodenal ulcer.

## References

[1] Amini A., Lopez R.A. Duodenal perforation.. http://www.ncbi.nlm.nih.gov/books/NBK553084/.

[2] Paspatis G.A., Dumonceau J.M., Barthet M. (2014). Diagnosis and management of iatrogenic endoscopic perforations: European Society of Gastrointestinal Endoscopy (ESGE) Position Statement. Endoscopy.

[3] Sultan M., Romero R., Evans J. (2022). Persistent gastro-cutaneous fistula closure with tack suture system in the setting of a severe esophageal stricture. VideoGIE.

[4] Zhang L.Y., Ghandour B., Bejjani M. (2022). Endoscopic full-thickness resection with through-the-scope suture closure for gastrointestinal stromal tumor. VideoGIE.

[5] Hayat M., Kadkhodayan K., Arain M.A. (2022). Successful management of a duodenal perforation using a through-the-scope suturing device after failed attempt at closure with an over-the-scope clip. VideoGIE.

[6] Hyun J.J., Kozarek R.A., Irani S.S. (2019). Endoscopic suturing of a large type I duodenal perforation. VideoGIE.

[7] Ge P.S., Thompson C.C. (2020). The use of the overstitch to close perforations and fistulas. Gastrointest Endosc Clin N Am.

[8] Kantsevoy S.V., Bitner M., Hajiyeva G. (2016). Endoscopic management of colonic perforations: clips versus suturing closure (with videos). Gastrointest Endosc.

